# Microstructure and Water Retention Kinetics in Autogenous Cured Self-Compacting Concrete Blends Using Super Absorbent Polymer

**DOI:** 10.3390/polym15183720

**Published:** 2023-09-11

**Authors:** Lija Rajamony Laila, Aarthi Karmegam, Siva Avudaiappan, Erick I. Saavedra Flores

**Affiliations:** 1 Department of Civil Engineering, KCG College of Technology, Chennai 600097, India; 2 Department of Civil Engineering, Government College of Engineering, Bodinayakkanur 625582, India; aarthi.karmegam@gmail.com; 3 Departamento de Ingeniería Civil, Universidad de Concepción, Concepción 4070386, Chile; savudaiappan@udec.cl; 4 Centro Nacional de Excelencia para la Industria de la Madera (CENAMAD), Pontificia Universidad Católica de Chile, Av. Vicuña Mackenna 4860, Santiago 8330024, Chile; 5 Departamento de Ingeniería en Obras Civiles, Universidad de Santiago de Chile, Av. Ecuador 3659, Estación Central, Santiago 9170022, Chile; erick.saavedra@usach.cl

**Keywords:** autogenous cured self-compacting concrete, water mitigation, super absorbent polymer, granite pulver, microstructural characteristics

## Abstract

This research aimed to determine how a super absorbent polymer affects the microstructural characteristics and water retention kinetics of a new composite made by substituting granite pulver (GP) and fly ash (FA) for cement. Understanding the mechanics of water movement is crucial for comprehending the effectiveness of autogenous curing. Several experiments were conducted to analyze the water mitigation kinetics of super absorbent polymer (SAP) in the hydrating cement paste of autogenous cured self-compacting concrete (GP-ACSSC) mixtures. In the first hours following casting, water sorptivity, water retention, and hydration tests were carried out. The effects of various concentrations of SAP and GP, which was utilized as an alternative cement for the production of sustainable concrete that leads to reduction in carbon footprint, on the autogenous cured self-compacting concrete with reference to the abovementioned properties were explored. The investigation showed that releasing the curing water at a young age, even around the beginning of hydration, allowed homogenous and almost immediate distribution of water across the full cured paste volume, which improved the water retention kinetics. Compared to the control mixtures, the addition of SAP up to 0.6% and the substitution of cement with GP up to 15% had favorable impacts on all water kinetics parameters.

## 1. Introduction

Concrete is a prevalent type of manufactured substance, with a global output varying around 35 to 53 billion tons in 2014 (estimated based on cement manufacturing, which contributes 8% to 12% of the manufacture of concrete) [1,2]. The widespread utilization of concrete has a number of disadvantages. It significantly affects the ecosystem. Cement production accounts for 5% to 7% of global carbon dioxide (CO_2_) emissions [3,4]. In fact, for every ton of cement produced, burning limestone causes CaCO_3_ to transform into calcium oxide (CaO), which then releases CO_2_ and results in the release of 1 ton of CO_2_. Additionally, the manufacture of cement results in a rise in the carbon footprint, which is a significant problem that threatens the viability of the ecosystem [5]. Several options are conceivable to address this issue, including the use of alternative cementitious substances such as fly ash and silica fumes to cut down on the quantity of Portland cement required [1,5,6,7,8,9,10]. The ability of structures to withstand chemical attacks, weathering, and abrasion resistance of concrete blends is a major problem when these alternative cementitious ingredients are used [11]. Furthermore, shrinkage, freeze/thawing, aggressive substances, and alkali–silica interactions all cause concrete to deteriorate over time. Drying and self-desiccation shrinkage can induce early-age cracking. During the curing process, fresh concrete may experience cycles of shrinkage and expansion, resulting in differential stress [12]. This tendency might lead to the creation of cracks. Curing the concrete allows for a reduction in transpiration of water and a surge in cement hydration [13]. Concrete with substantial strength has a low water-to-cement concentration (0.4), so there will not be enough integrating water for complete hydration. The capillary water is used first, and the binder then reacts with the larger covalently bonded fluid gel [14]. The molecular structure becomes denser, resulting in reaction products that are smaller relative to the quantity. Shrinkage and cracking may develop from the cementitious matrix drying out on its own due to reacting substances and the shortage of external moisture to replenish the voids [15,16,17,18]. When surface moisture evaporates faster than it can be replaced by the interior water, plastic shrinkage occurs, leading to the surface becoming smaller than the massive substance. Chemical diminution brings about autogenous shrinkage or changes in the concentration of cementitious material, mortar, or concrete. The paste loses homogeneous volume when the internal relative humidity falls below a particular threshold. This is not caused by thermal factors, external stresses or constraints, or moisture loss into the environment [19]. Investigation on the effect of paste volume on SCC shrinkage [20] has demonstrated that SCC shrinkage is significantly greater than that of conventionally vibrated concrete.

Recently, an autogenous curing technique based on super absorbent polymer (SAP), which is also a water entrainment, has been used as an effective means of preventing self-desiccation [21,22] and shrinking due to self-desiccation (for example, [21,22,23,24]). SAPs are designed to act as internal water reservoirs and should be evenly dispersed throughout the concrete. After being moistened while combining, they disperse water to the paste around them. SAPs can be utilized to provide water curing in situations where the material’s areas that are far from the cured surface cannot be accessed by externally supplied curing water due to reduced proportion of water-to-cement (w/c) combinations along with extremely fine structures. However, internally cured materials must also handle the problems with water mobility inside the hard cement paste’s matrix. In addition to the issue of having enough entrained water, it is essential that the entrapped water is available as the entire quantity of the cured mixture [21]. In comparison with the usual tests for self-desiccation and hydration, the water must move over short distances in the area of the reservoir paste in a faster way. Therefore, the spatial distribution of reservoirs [25,26] and the water’s ability to move around in the curing cement paste are two factors that need to be addressed in order to determine the dimensions of the cured volume [23,27]. The latter problem is mostly addressed in this work.

## 2. Materials and Methods

**Figure 1:**
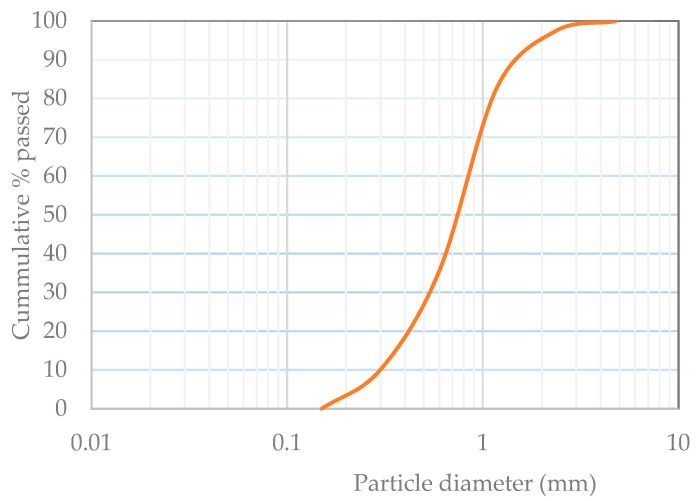
Grain size distribution.

Ordinary Portland cement (OPC) of Grade 53 in accordance with IS 12269-1987 [28] was employed to prepare the GP-ACSCC combinations. Using the procedure prescribed in IS 12269-1987 [28] and IS 4031-1988 [29], the OPC’s physical properties and mineralogical constitution were determined, respectively. The standard homogeneity was 31%, the specific gravity was 3.10, and the particle size measured by Blaine’s air permeability according to IS 4031-1988 [29] was 354 m^2^/kg. Chengalpattu River close to Chennai, India, has a stream sand with a bulk density of 1610 kg/m^3^, which was employed as aggregates to prepare the GP-ACSCC mixtures. The specific gravity was 2.66, the fineness modulus was 2.67, and Zone II spanned from 4.75 mm to 150 µm. Figure 1 depicts the fine aggregate grain size distribution.

In concrete compositions, coarse particles are the most durable and least porous. In this experiment, the control and GP-ACSCC mixtures were made using coarse aggregates with a bulk density of 1160 kg/m^3^. In accordance with the European Federation of Specialist Construction Chemicals and Concrete Systems (EFNARC) [30], angular coarse particles with a maximum size of 12 mm were used in this investigation to prevent aggregate obstruction when pouring the concrete. According to IS 2386-1963 [31], the coarse aggregates used in this work had 2.86 specific gravity and a fineness modulus of 6.88. For the purpose of this investigation, fly ash of class F was employed, with 2.42 noted as the specific gravity. The chemical properties were measured according to the IS 3812-2003 [32] method. Utilizing X-ray diffraction (XRD, Bruker, Germany) and scanning electron microscopy with energy-dispersive spectroscopy (SEM-EDAX, Bruker, Germany) studies, the mineralogical components of the fly ash were identified. The dispersion and fineness characteristics of fly ash enhances the strength and durability properties of concrete blends. Numerous studies have shown that adding fly ash, in particular, results in increased resistance to chloride penetration. Granite pulver (GP) from India’s Vellore area was employed as a cement substitute in the concrete blends in the experimental investigation. In order to cut and process, granite tiles were used and GP was produced as a by-product. The GP’s physical and chemical characteristics were investigated. The GP had a specific gravity of 2.57, and its mineralogical makeup was ascertained with studies using XRD and SEM-EDAX. The particle fineness of the GP was assessed using IS 4031-1988 [29], and the particle fineness of the GP employed in this investigation was found to be up to 305 m^2^/kg. The super absorbent polymer (SAP) is a network of hydrophilic polymers that has a high ability to absorb water. SAPs hydrate when they come into contact with water and form a swelling gel polymer structure. SAPs and water interact to create pores that circulate effectively in cementitious matrices of materials. SAPs can work as self-curing agents. SAPs are mixed with GP-ACSCC mixtures at varying concentrations (0.1 to 1 percent) in accordance with the manufacturer’s guidelines because they are anticipated to work as self-curing agents. In this investigation, white-appearing SAPs with an average bulk density of 0.85 g/cm^3^ and an absorption rate of 800 g/g of water were used. Superplasticizer is a chemical ingredient that makes concrete mixtures easier to work with without adding more water. The FOSROC Company’s Conplast SP430 superplasticizer was used to combine with the concrete in this case. As a high range water reduction additive, it complies with IS 9103-1999 [33].

In this investigation, M30 grade concrete was employed with a mix ratio of 1:2.12:1.75 (powder: fine aggregates: coarse aggregate). Here, cement, fly ash, and granite pulver refer to powder content. In the mixtures, 5%, 10%, 15%, and 20% of the cement content was replaced by granite pulver by mass with the addition of 0.1%, 0.2%, 0.3%, 0.4%, 0.5%, 0.6%, 0.8%, and 1.0% of super absorbent polymers by volume fraction. Lija et al. [34], Deepankarkumar et al. [35], and Karmegam and Kalidass [36,37] emphasized the importance of particle packing and specific gravity in determining the mix proportion.

**Figure 2:**
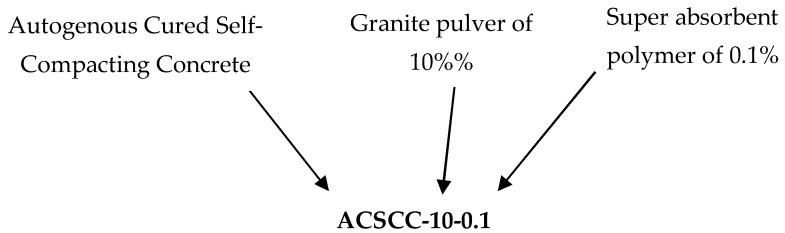
Specimen labeling.

To distinguish the concrete mixtures, the samples were properly labeled. The autogenous cured self-compacting concrete (ACSCC-X-Y) design mixture with GP content of X% and SAP content of Y% is referred to in this article. A sample labeling for ACSCC-10-0.1, for instance, is given below (Figure 2)

### 2.1. Chemical and Microstructural Properties

GP, fly ash, and cement microstructural characteristics were investigated under SEM, and components were determined with the help of EDAX data. The Match Crystal Impact software helped us find the mineral compositions of GP, fly ash, and cement. The matching qualities were produced by the samples that underwent XRD analysis to determine items and their compositions.

### 2.2. Water Retention Properties

#### 2.2.1. Weight Loss

Concrete weight loss was evaluated by filling 1.5 L polypropylene containers with an interior circumference of 120 mm and an elevation of 130 mm with concrete. A steady temperature of about 25 degrees Celsius and a humidity of about 65 percent were used to maintain the container. After casting, the container’s weight was taken at different points in time to evaluate the weight reduction over time. The measurement of weight was recorded until the specimen turned 28 days old. Two samples were used for each blend, and the average values were used for further discussion.

#### 2.2.2. Internal Relative Humidity

Each mixture yielded a cube specimen with dimensions of 150 × 150 × 150 mm. For a 24 h curing period, the cubes were left in the molds. Following demolding, each cube had an opening drilled from its top face with a radius of 10 mm and a depth of 100 mm. Air jet was used to clean the hole. It took away any loose debris. A rubber stopper was used to close the hole. The cube was then shut off from the rest of the world with wax. Up until the samples were 91 days old, the relative humidity inside the cube was recorded employing a computer-controlled relative humidity probe that was sealed inside the concrete block with a one-hole rubber stopper. The probe needed to be kept inside a cavity for two to three hours prior to performing the readings. It took about 20 to 30 s for the relative humidity value to become normal. The perforations were sealed with a solid rubber stopper after the internal relative humidity had been measured using probes. Duplicate specimens of each mixture were created, and the average outcomes were used for analyses.

### 2.3. Nonevaporable Water

The nonevaporable moisture content for every sample for each combination was recorded over a period of up to 28 days. The specimens were dried by exposure in air. By screening the crushed concrete sample in order to remove coarse particles, a sample cement paste needed to be created. A concrete specimen was crushed at each age from each mix. To avoid hydration, prior to testing, the specimens were placed in propanol. The quantity of nonevaporable water was calculated using the loss of weight following combustion at 105 °C in a muffle furnace. The difference between the weight lost and the specimens’ initial weight (g/g) helped to calculate nonevaporable water. Duplicate samples were used for each mixture and each test age, and the mean results were employed for analyses.

### 2.4. Sorptivity 

The water sorptivity test was carried out to assess the degree of consumption of hydraulic cement-based blends. The test pieces were discs that had been cut from cylinders with 50 mm radius and 50 mm height. The specimens were dried for 24 h at 110 °C in a combustion chamber, followed by another 24 h of cooling while still dry. Utilizing a support in the shape of a circle, one plane of the specimen was close to the water, which was 5 mm deep during the test. By maintaining the exterior water level between 1 and 3 mm higher than the surface of the support while using the frame as a support, it was feasible to maintain uninterrupted contact with water over the test duration without altering the water depth. Electric vinyl tape was used to seal the test specimens’ sides so that the concrete specimen would only flow in one direction. A total time of 25 min was spent recording the specimen’s weight at predetermined intervals [38,39]. The sorptivity test was carried out on triplicate specimens for each combination at 28 and 56 days of age.

## 3. Results and Discussions

### 3.1. Chemical and Microstructural Properties

**Figure 3:**
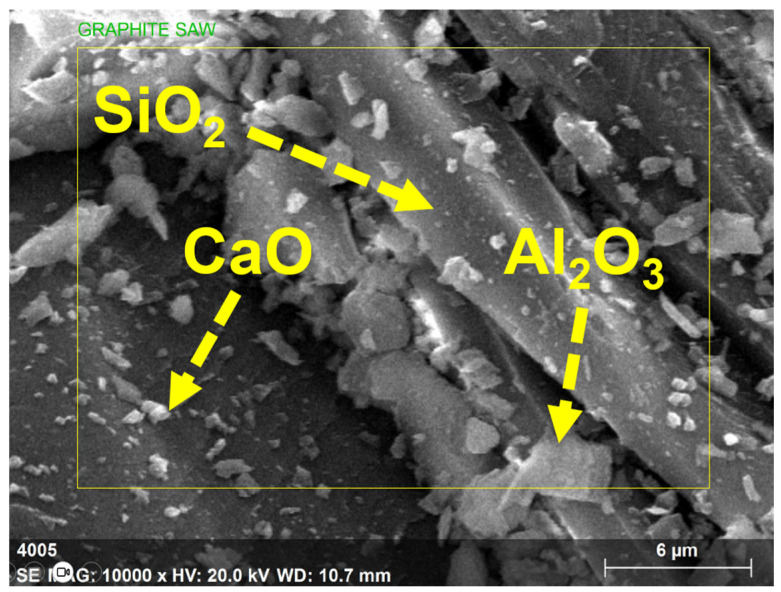
SEM image of GP.

Cement, fly ash, and GP samples’ morphology was examined using SEM-EDAX. The picture in Figure 3 at a scale of 6 micrometers (µm) was created through testing and demonstrates the extremely changeable size of GP particles. The GP particles’ surfaces were angular, irregular, and had a high degree of surface roughness. The results of GP in Figure 3 reveal a high concentration of silica, calcium, and alumina, all of which support pozzolanic activity.

**Figure 4:**
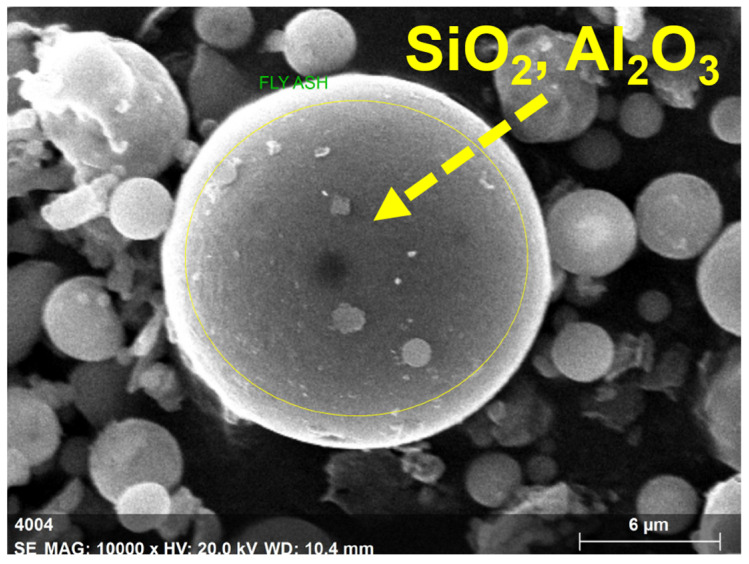
Microscopic image of fly ash.

**Figure 5:**
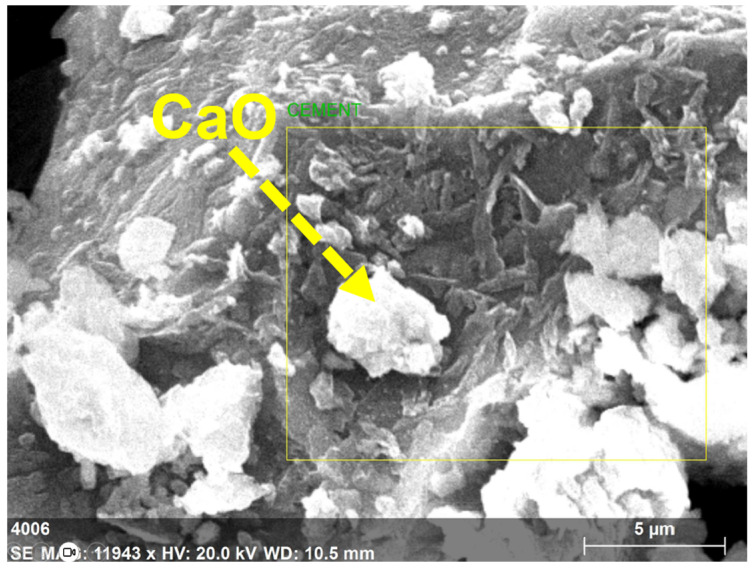
Microscopic image of cement.

The fly ash particles, shown in Figure 4 in a 6 µm scale picture, were somewhat smaller than the GP particles. The spherical form of the fly ash particles, as shown in Figure 4, could help the enhancement of flow properties of the GP-ACSCC mixtures. GP particles have lower values than fly ash particles with regard to fineness and specific surface. Additionally, Figure 4 depicts the indication of calcium, silica, iron, and alumina. The element details in cement are shown by the SEM findings and the 5 µm scale picture of cement grains in Figure 5. This image demonstrates the uneven morphology of the cement grains. In their investigations, Lija et al. [34,40], Karmegam and Kalidass [36], and I. Mormol et al. [41] all found the same morphology and existence of components.

**Figure 6:**
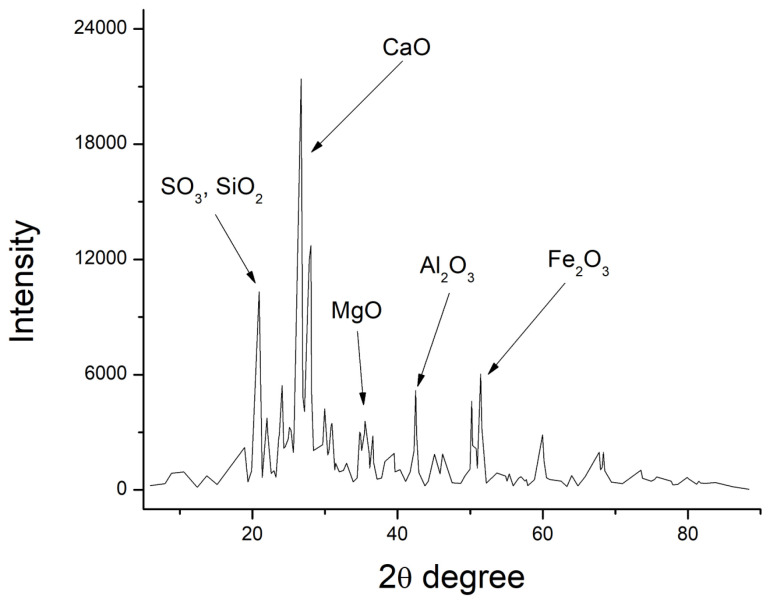
X-ray diffraction of GP.

**Figure 7:**
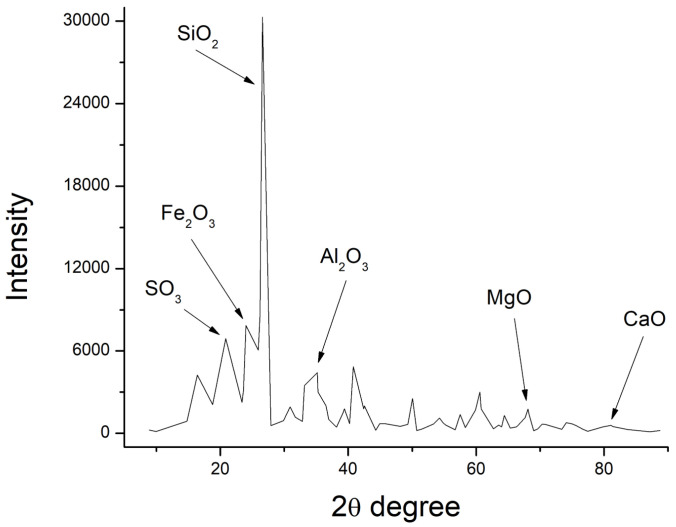
X-ray diffraction of fly ash.

**Figure 8:**
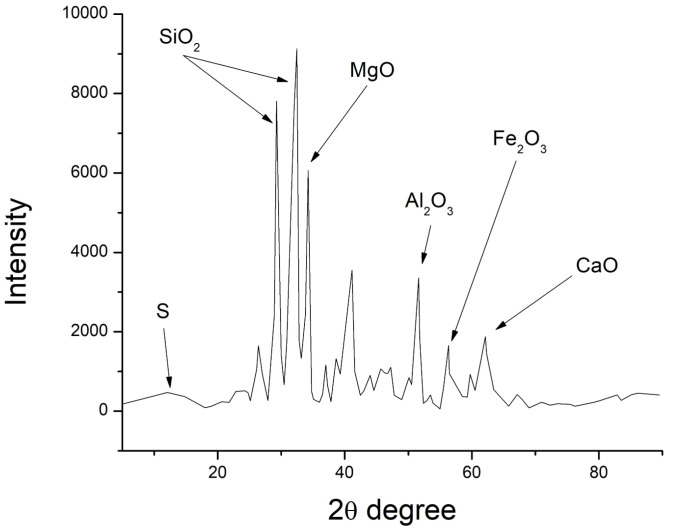
X-ray diffraction of cement.

Match Crystal Impact software was used to help evaluate the XRD patterns, as shown in Figure 6, Figure 7 and Figure 8, and the chemical characteristics of GP, fly ash, and cement were examined. The research verified the presence of calcium oxide, iron, silica, and alumina concentration in fly ash and GP. Fly ash contained a significant amount of quartz (SiO_2_) (52.6 percent). The second most abundant mineral in the fly ash was alumina (Al_2_O_3_), which made up 38.4 percent of the total. Similarly, the largest percentage (50.3%) of silicon dioxide (SiO_2_) was identified in GP, which was also present in fly ash.

According to XRD data, the most prevalent components in GP and fly ash specimens were silicon, aluminum, iron, oxygen, and calcium. Due to their possible pozzolanic and semicementitious properties, silica, and alumina have a positive impact on GP and fly ash, making them good cement alternatives. The reactive silica content of fly ash and GP as mineral admixtures was higher than the minimum criterion set forth by Karmegam and Kalidass [36] in their work.

**Figure 9:**
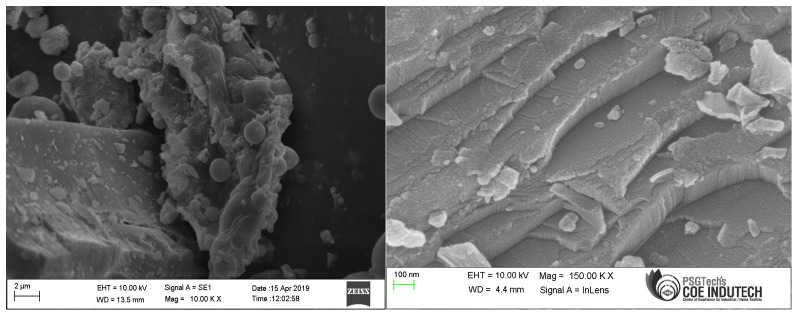
Microscopic image of GP.

**Figure 10:**
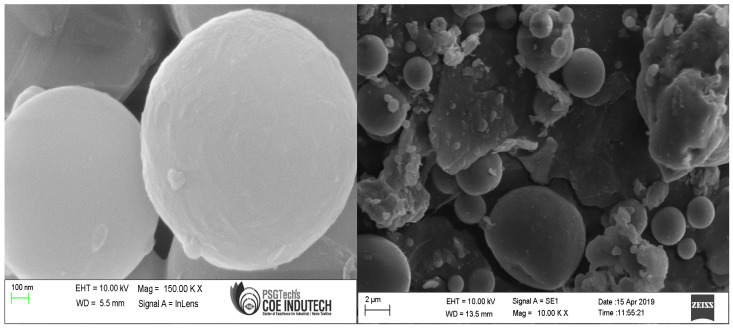
Microscopic image of fly ash.

**Figure 11:**
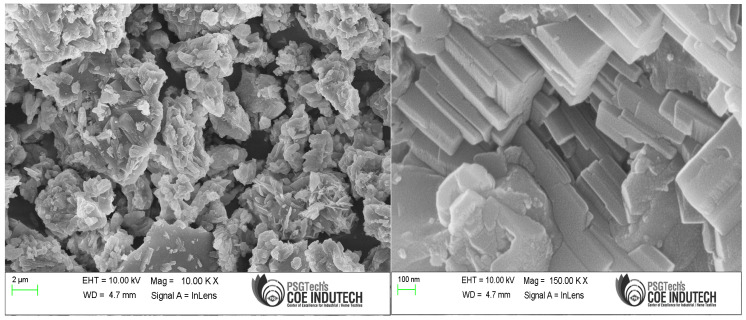
Microscopic image of cement.

The images in Figure 9, Figure 10 and Figure 11 show the particle distribution for fly ash, GP, and cement specimens at a size of 100 nm. According to IS 3812-2003 [32], the three oxides added together (ferric oxide (Fe_2_O_3_), alumina (Al_2_O_3_), and silica (SiO_2_)) should be greater than 70% in order for natural pozzolans to qualify as Class N natural pozzolans that have been calcined and are suitable for employing in the blend. The existence of silica, ferric oxide, and aluminum oxide meets the prerequisites, as XRD studies have verified. GP can be used as a cement alternative according to the chemical and microstructural examinations. The conclusion implies that the primary morphological patterns are rather widespread in cement and GP samples.

**Figure 12:**
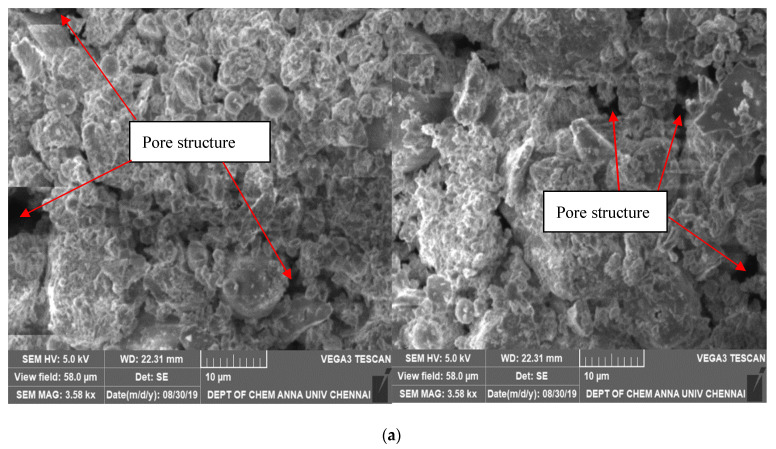

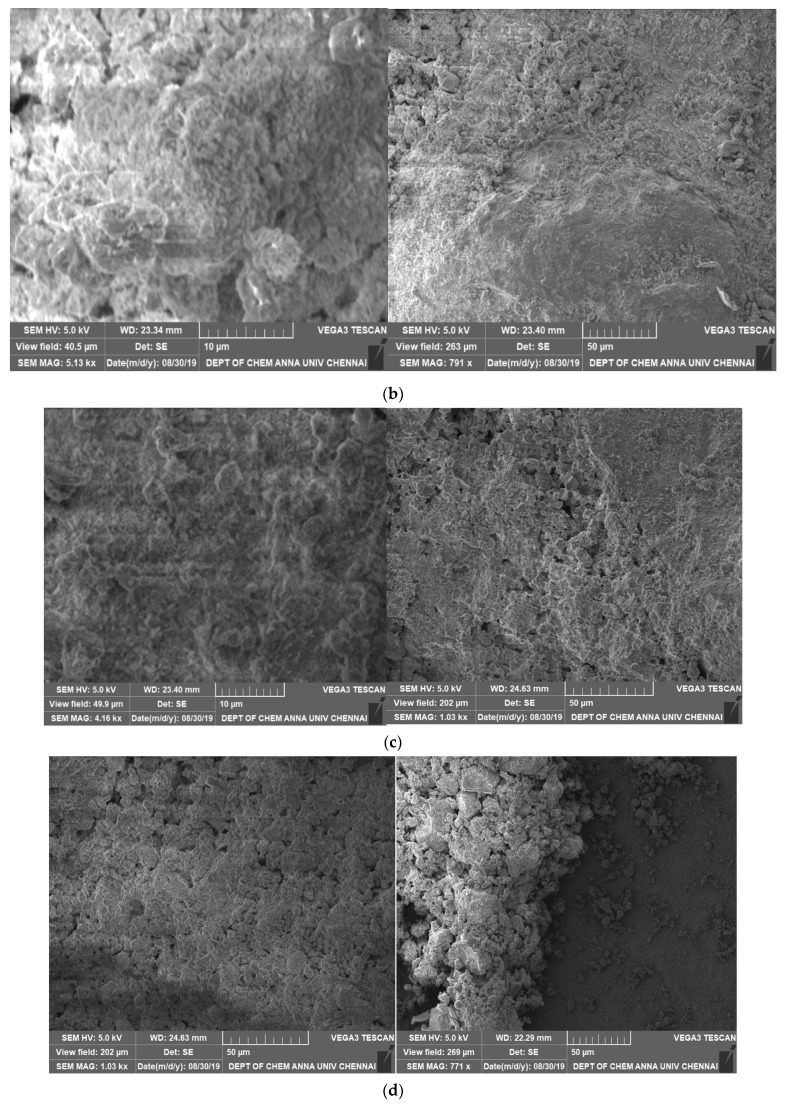
(**a**) Microscopic image of control specimen at 28 days of age. (**b**) Microscopic image of ACSCC-10-0.4 at 28 days of age. (**c**) Microscopic image of ACSCC-15-0.3 at 28 days of age. (**d**) SEM micrographs of ACSCC-20-0.6 at 28 days of age.

Because the quantity of water in concrete impacts how the microstructure develops and hardens, the water kinetics in specimens with SAPs are crucial to understanding the microstructural features and the moist mechanisms involved. The microstructural evolution of a mixture is determined by its hydration. Pastes with SAPs have more capillary porosity if additional water is added in order to account for the decreased workability [42]. The control specimen’s microstructure can be seen in the images at 10 and 50 µm scales in Figure 12a. There are more visible pore structures with relatively large diameter and less homogeneity in this image. This is because of the weak surface hydration response and less interface interactions within the concrete matrix. The microstructure of ACSCC-10-0.4 and ACSCC-15-0.3 at 10 and 50 µm scales is given in Figure 12b,c which shows they are the most homogenous of all the GP-ACSCC mixtures. In the image, the gel formed from calcium silica hydration (C-S-H) is more visible. It is clear that the GP-ACSCC combination concrete is tightly packed, homogenous, and has lesser pore structures in comparison to the control specimen, leading to a stronger water retention property and lower shrinkage, as illustrated in Figure 12b,c. Beyond 0.6% of SAP, the void increases and thus lowers the homogeneity of the concrete, as demonstrated in Figure 12d. According to the findings of B. Craeye et al. [43], SAP addition is responsible for the homogeneity in GP-ACSCC combinations. B. Craeye et al. [43] claimed that adding SAP to concrete initially promotes the emergence of pore structures, but it also hastens the hydration process by supplying internal water and helps form the hydrated product, which fills the pores.

Variations in the pore dimensions can have a substantial impact on the mobility of cement pastes. SAP-produced macropores need to have a lesser effect on cement paste’s transport properties as long as they create separate, independent voids. In addition, a matrix with less porosity should, in theory, have less transport properties and more durable cement-based products. This idea was backed by research on the permeability and capillary attraction of concrete with SAP [44].

### 3.2. Water Retention Properties

#### 3.2.1. Weight Loss

**Figure 13:**
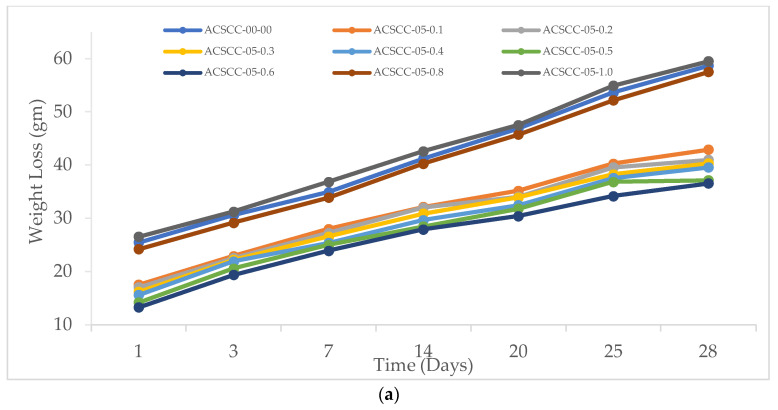

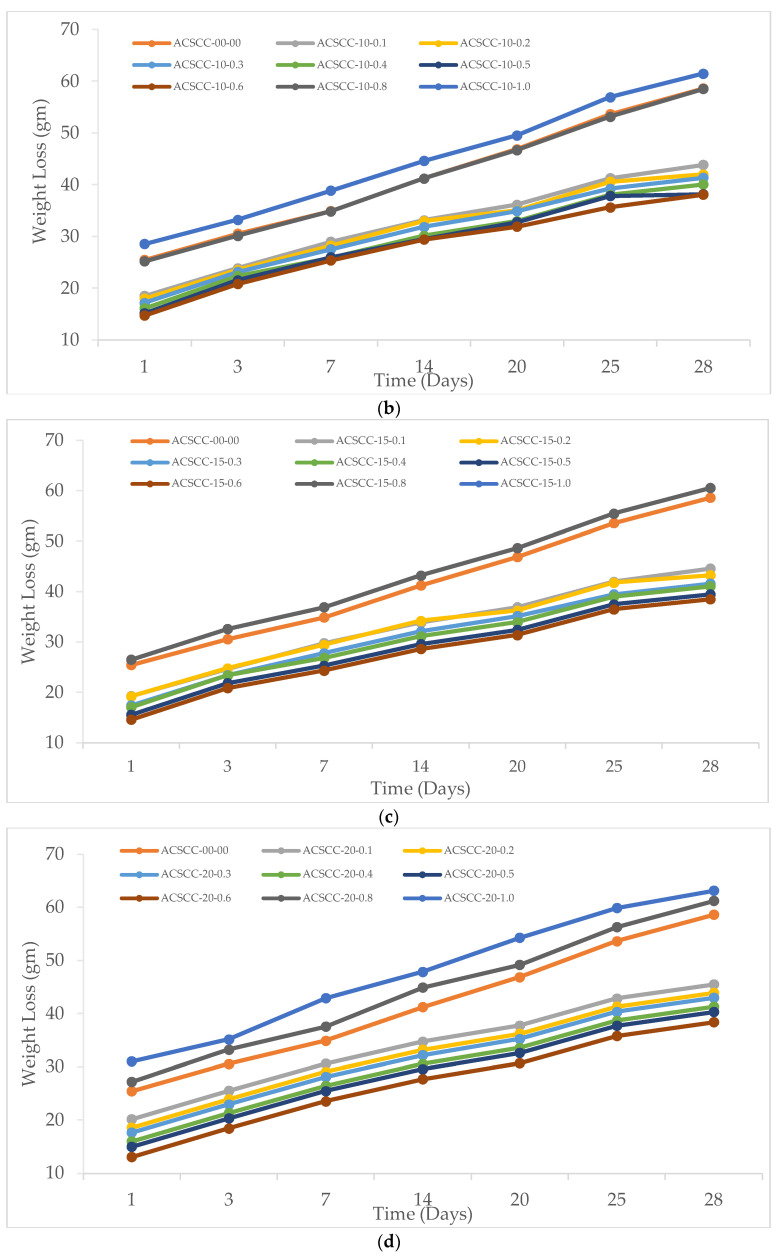
Weight loss of GP-ACSCC mixtures with time. (**a**) 5% GP, (**b**) 10% GP, (**c**) 15% GP, and (**d**) 20% GP.

It was discovered that the GP-ACSCC blends lost less weight over time as a result of moisture evaporation than the traditional mixtures. This suggests that GP-ACSCC mixtures retain water more effectively. Figure 13 depicts the weight decrease over time for each blend. In comparison to concrete mixtures with SAP, weight loss for concrete mixtures without SAP was larger. Furthermore, GP-ACSCC blends with addition of SAP above 0.6% lost weight more quickly than those with SAP additions below 0.6%. Figure 13 makes it obvious that the weight loss of the specimens decreases as GP and SAP content increases, demonstrating that water retention increases with the addition of a high percentage of GP and SAP to GP-ACSCC blends.

#### 3.2.2. Internal Relative Humidity

**Figure 14:**
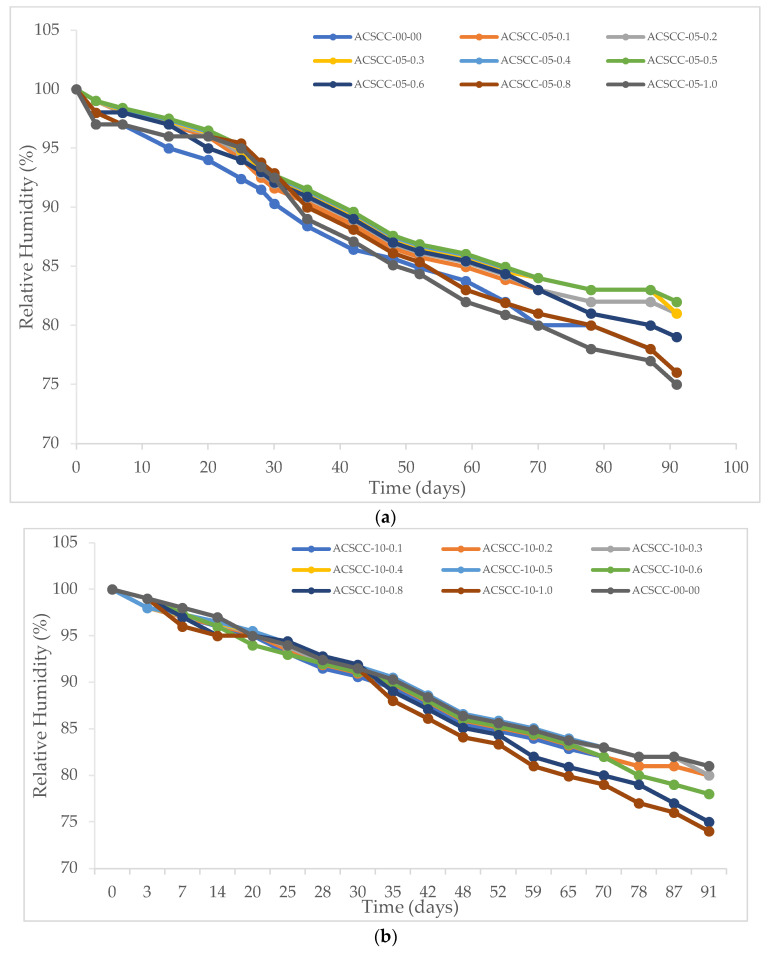

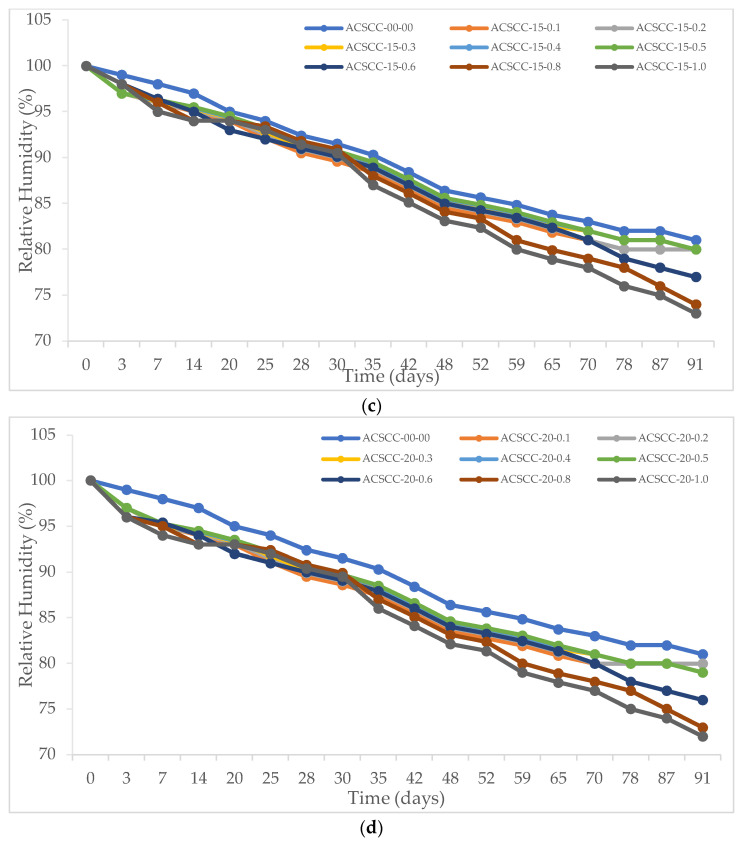
Internal relative humidity of GP-ACSCC mixtures with time. (**a**) 5% GP, (**b**) 10% GP, (**c**) 15% GP, and (**d**) 20% GP.

Table 1 and Figure 14 depict internal relative humidity over time of GP-ACSCC combinations as well as standard concrete. Concrete’s internal relative humidity was significantly changed due to kinetics of internal water, regardless of whether GP-ACSCC blends or standard mixtures were used. This finding is consistent with those made previously for conventional concrete mixtures [45,46]. For practically all GP-ACSCC combinations with SAP dosages up to 0.6%, the internal relative humidity was slightly above 80% until 91 days of age, while it was below 80% for GP-ACSCC mixtures with SAP content more than 0.6%. According to the findings, standard mixtures exhibited higher self-desiccation than GP-ACSCC combinations, which might have an impact on how hydrated the cement is. According to reports, the process of hydration is severely inhibited by a decrease in internal relative humidity [47]. Thus, by maintaining relative humidity at greater levels, SAP will enable a higher level of ultimate hydration to be anticipated.

### 3.3. Nonevaporable Water

**Figure 15:**
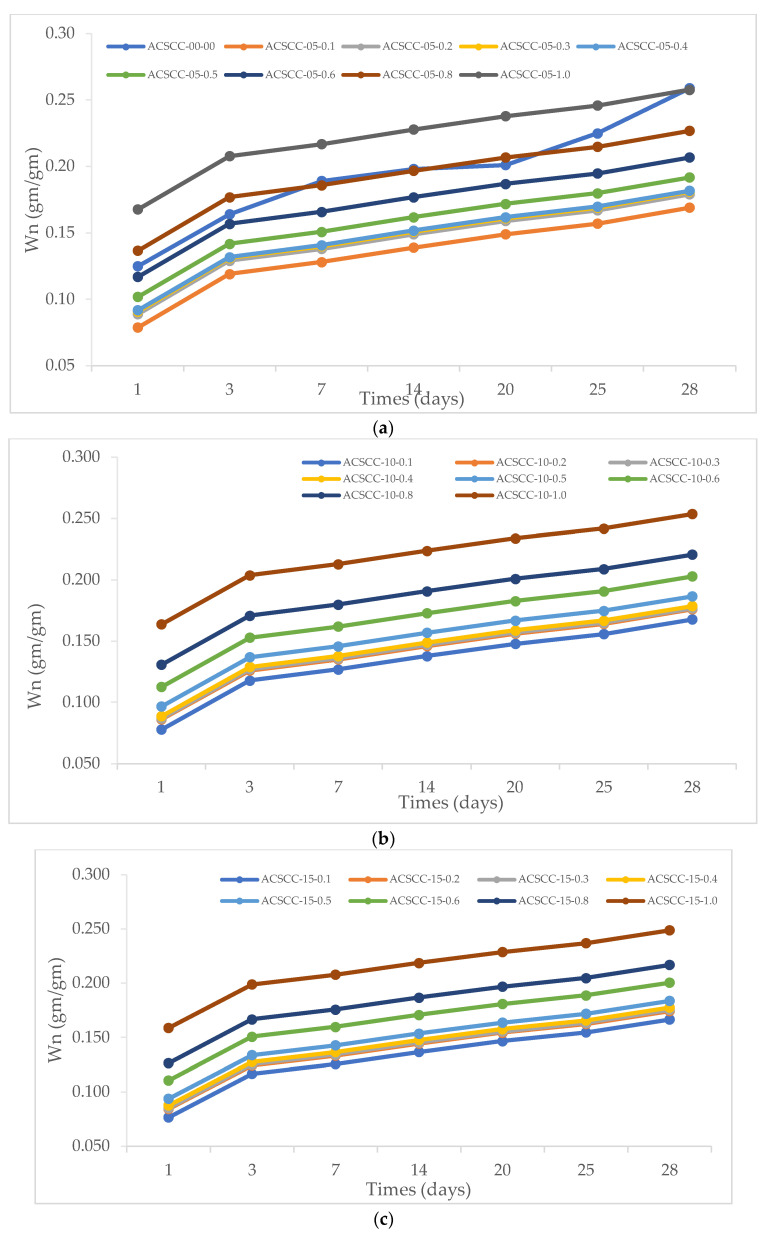

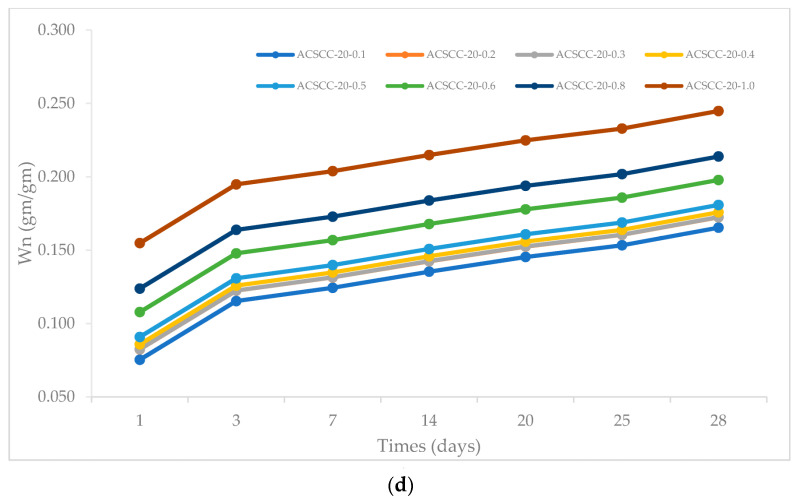
Nonevaporable water of GP-ACSCC mixtures with time. (**a**) 5% GP, (**b**) 10% GP, (**c**) 15% GP, and (**d**) 20% GP.

Nonevaporable water (Wn), taken using uncapped samples (i.e., under drying circumstances) at various intervals for GP-ACSCC combinations and conventional concrete mixtures, are shown in Table 2 and Figure 15. The GP-ACSCC mixture’s ability to keep water within itself resulted in larger concentrations of nonevaporable water and, as predicted, more concentrations of hydration. The results were influenced by the mix proportions, which were established by internal relative humidity and weight loss assessments. Jensen and Hansen [21,22] had already theoretically predicted the accelerating cement hydration in internal curing using SAP, and Lura et al. [24] had reported experimental findings that support this theory. Internal curing with SAP resulted in a higher degree of cement hydration in the end, as was also discovered in [48]. Zhutovsky and Kovler [49] demonstrated that SAP’s impact on cement hydration is significant. This is in line with the findings mentioned in Figure 15. The process of hydration is severely inhibited by a decrease in internal relative humidity, particularly when it falls below 80% [47]. Thus, by maintaining relative humidity at greater levels, SAP will enable a higher level of ultimate hydration, which in line with the results mentioned in Figure 15.

### 3.4. Sorptivity

**Figure 16:**
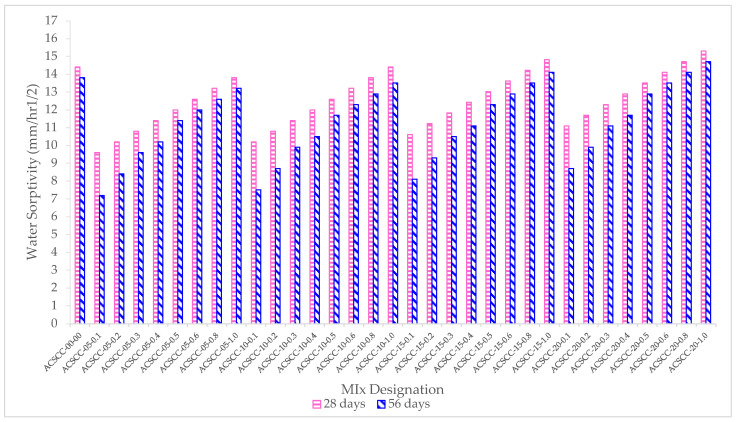
Water sorptivity of GP-ACSCC mixtures at various ages.

Concrete’s sorptivity was examined at 28 and 56 days to see how self-curing affects the formation of capillary holes and water suction. Table 3 and Figure 16 show the sorptivity for regular concrete and GP-ACSCC mixtures under two different curing regimes at 28 and 56 days of age. At both ages, for the consistently water-cured state, it was discovered that the normal concrete mixture without SAP had higher water sorptivity values. It was evident from the findings that the GP-ACSCC blends had higher water sorptivity values than the water-cured standard mixture. This supports the findings for other water retention kinetics properties. The sorptivity values of both the GP-ACSCC blends and the water-cured concrete mixtures were observed to decrease over time, but the drop for the water-cured mixture was greater than that for the GP-ACSCC blends. This may be explained by the continued hydration in both mixtures; however, in the case of GP-ACSCC blends, the effect was insignificant in reducing the volume of enormous pores. The decreased pace of hydration may be to blame for this.

As per the guidelines of ASTM C1585 [50], the sorptivity value obtained for most of the GP-ACSCC combinations was classified as “very good” and “good”. This shows that GP-ACSCC mixtures have poorer pores. It is evident from Figure 16 that the majority of the GP-ACSCC blends fell in the “very good” and “good” criteria; however, the introduction of SAP beyond 0.5 percent accelerated the sorptivity value, which fell in the “poor” criteria as per ASTM C1585 [50]. Among all the 32 GP-ACSCC blends, two mixtures (ACSCC-05-0.1 and ACSCC-15-0.1) had the lowest sorptivity values (Figure 16).

## 4. Conclusions

This study focused on the water retention kinetics of a unique GP-ACSCC mixture. The following observations can be derived from the findings:

1. The core mineral and morphological features in the GP and cement specimens were comparable according to SEM-EDAX analysis.
2. The microstructural studies showed that SAP addition up to 0.6% sped up the hydration process by supplying internal water and helped form the hydrated product.
3. The inclusion of SAP and GP finally caused the weight reduction to halt. On the other hand, with time, when SAP addition was more than 0.6%, the weight loss quickened. For the GP-ACSCC combinations, weight loss was less than that for the control mixture.
4. A significant increase in relative humidity was noticed for 91 days compared to the conventional mixture for almost all GP-ACSCC mixtures.
5. SAP addition up to 0.6% demonstrated a considerable increase in water retention qualities among the 32 GP-ACSCC mixtures.
6. Compared to control mixtures, the addition of SAP up to 0.6% and the substitution of cement with GP up to 15% had favorable impacts on all water kinetics parameters.
7. Larger quantities of nonevaporable water were produced as a result of the GP-ACSCC mixture’s capacity to store water, which in turn led to higher levels of hydration.
8. Both the GP-ACSCC blends and the conventional concrete mixture sorptivity values were observed to decrease over time, while most of the GP-ACSCC blends fell under the “very good” and “good” criteria as per the code provision.
9. If SAP is evenly distributed across the hydration zones, it will more effectively function as an internal curing substance. The GP-ACSCC mixture’s small particle size and structure enabled the SAP to be distributed evenly and improved the pore structure. Self-desiccation was prevented by the available curing water at the small reservoirs of the super absorbent polymers. The high water retention property and improved kinetics of water was proven by the increase in nonevaporable water, increase in relative humidity, and decrease in percentage of weight loss. It was obvious that the GP-ACSCC combination concrete was more densely packed, homogeneous, and had a more refined pore structure in comparison with the control specimen. This produced a higher water retention property.

## Figures and Tables

**Table 1:** Internal relative humidity of GP-ACSCC mixtures.

Mixture Designation	Relative Humidity (%)																	
Days	0	3	7	14	20	25	28	30	35	42	48	52	59	65	70	78	87	91
ACSCC-00-00	100	98	97	95	94	92	92	90	88	86	86	85	84	82	80	80	78	77
ACSCC-05-0.1	100	99	98	97	96	94	93	92	90	89	87	86	85	84	83	82	82	81
ACSCC-05-0.2	100	99	98	97	96	95	93	92	91	89	87	86	85	84	83	82	82	81
ACSCC-05-0.3	100	99	98	97	96	95	93	92	91	89	87	86	86	85	84	83	83	81
ACSCC-05-0.4	100	99	98	97	96	95	94	93	91	90	88	87	86	85	84	83	83	82
ACSCC-05-0.5	100	99	98	98	97	95	94	93	92	90	88	87	86	85	84	83	83	82
ACSCC-05-0.6	100	98	98	97	95	94	93	92	91	89	87	86	85	84	83	81	80	79
ACSCC-05-0.8	100	98	97	96	96	95	94	93	90	88	86	85	83	82	81	80	78	76
ACSCC-05-1.0	100	97	97	96	96	95	93	93	89	87	85	84	82	81	80	78	77	75
ACSCC-10-0.1	100	99	98	97	95	94	92	92	90	88	86	86	85	84	83	82	82	81
ACSCC-10-0.2	100	98	97	96	95	93	92	91	89	88	86	85	84	83	82	81	81	80
ACSCC-10-0.3	100	98	97	96	95	94	92	91	90	88	86	85	84	83	82	81	81	80
ACSCC-10-0.4	100	98	97	96	95	94	92	91	90	88	86	85	85	84	83	82	82	80
ACSCC-10-0.5	100	98	97	96	95	94	93	92	90	89	87	86	85	84	83	82	82	81
ACSCC-10-0.6	100	98	97	97	96	94	93	92	91	89	87	86	85	84	83	82	82	81
ACSCC-10-0.8	100	99	97	96	94	93	92	91	90	88	86	85	84	83	82	80	79	78
ACSCC-10-1.0	100	99	97	95	95	94	93	92	89	87	85	84	82	81	80	79	77	75
ACSCC-15-0.1	100	99	98	97	95	94	92	92	90	88	86	86	85	84	83	82	82	81
ACSCC-15-0.2	100	97	96	95	94	92	91	90	88	87	85	84	83	82	81	80	80	80
ACSCC-15-0.3	100	97	96	95	94	93	91	90	89	87	85	84	83	82	81	80	80	80
ACSCC-15-0.4	100	97	96	95	94	93	91	90	89	87	85	84	84	83	82	81	81	80
ACSCC-15-0.5	100	97	96	95	94	93	92	91	89	88	86	85	84	83	82	81	81	80
ACSCC-15-0.6	100	97	96	96	95	93	92	91	90	88	86	85	84	83	82	81	81	80
ACSCC-15-0.8	100	98	96	95	93	92	91	90	89	87	85	84	83	82	81	79	78	77
ACSCC-15-1.0	100	98	96	94	94	93	92	91	88	86	84	83	81	80	79	78	76	74
ACSCC-20-0.1	100	99	98	97	95	94	92	92	90	88	86	86	85	84	83	82	82	81
ACSCC-20-0.2	100	97	95	94	93	91	90	89	87	86	84	83	82	81	80	80	80	80
ACSCC-20-0.3	100	97	95	94	93	92	90	89	88	86	84	83	82	81	80	80	80	80
ACSCC-20-0.4	100	97	95	94	93	92	90	89	88	86	84	83	83	82	81	80	80	79
ACSCC-20-0.5	100	97	95	94	93	92	91	90	88	87	85	84	83	82	81	80	80	79
ACSCC-20-0.6	100	97	95	95	94	92	91	90	89	87	85	84	83	82	81	80	80	79
ACSCC-20-0.8	100	96	95	94	92	91	90	89	88	86	84	83	82	81	80	78	77	76
ACSCC-20-1.0	100	96	95	93	93	92	91	90	87	85	83	82	80	79	78	77	75	73

**Table 2:** Hydration or nonevaporable water of GP-ACSCC mixtures.

Mixture Designation	Nonevaporable Water						
Days	1	3	7	14	20	25	28
ACSCC-00-00	0.070	0.110	0.120	0.130	0.145	0.150	0.168
ACSCC-05-0.1	0.079	0.119	0.128	0.139	0.149	0.157	0.169
ACSCC-05-0.2	0.089	0.129	0.138	0.149	0.159	0.167	0.179
ACSCC-05-0.3	0.091	0.131	0.140	0.151	0.161	0.169	0.181
ACSCC-05-0.4	0.092	0.132	0.141	0.152	0.162	0.170	0.182
ACSCC-05-0.5	0.102	0.142	0.151	0.162	0.172	0.180	0.192
ACSCC-05-0.6	0.117	0.157	0.166	0.177	0.187	0.195	0.207
ACSCC-05-0.8	0.137	0.177	0.186	0.197	0.207	0.215	0.227
ACSCC-05-1.0	0.168	0.208	0.217	0.228	0.238	0.246	0.258
ACSCC-10-0.1	0.078	0.118	0.127	0.138	0.148	0.156	0.168
ACSCC-10-0.2	0.086	0.126	0.135	0.146	0.156	0.164	0.176
ACSCC-10-0.3	0.087	0.127	0.136	0.147	0.157	0.165	0.177
ACSCC-10-0.4	0.089	0.129	0.138	0.149	0.159	0.167	0.179
ACSCC-10-0.5	0.097	0.137	0.146	0.157	0.167	0.175	0.187
ACSCC-10-0.6	0.113	0.153	0.162	0.173	0.183	0.191	0.203
ACSCC-10-0.8	0.131	0.171	0.180	0.191	0.201	0.209	0.221
ACSCC-10-1.0	0.164	0.204	0.213	0.224	0.234	0.242	0.254
ACSCC-15-0.1	0.077	0.117	0.126	0.137	0.147	0.155	0.167
ACSCC-15-0.2	0.085	0.125	0.134	0.145	0.155	0.163	0.175
ACSCC-15-0.3	0.086	0.126	0.135	0.146	0.156	0.164	0.176
ACSCC-15-0.4	0.088	0.128	0.137	0.148	0.158	0.166	0.178
ACSCC-15-0.5	0.094	0.134	0.143	0.154	0.164	0.172	0.184
ACSCC-15-0.6	0.111	0.151	0.160	0.171	0.181	0.189	0.201
ACSCC-15-0.8	0.127	0.167	0.176	0.187	0.197	0.205	0.217
ACSCC-15-1.0	0.159	0.199	0.208	0.219	0.229	0.237	0.249
ACSCC-20-0.1	0.075	0.115	0.124	0.135	0.145	0.153	0.165
ACSCC-20-0.2	0.082	0.122	0.131	0.142	0.152	0.160	0.172
ACSCC-20-0.3	0.083	0.123	0.132	0.143	0.153	0.161	0.173
ACSCC-20-0.4	0.086	0.126	0.135	0.146	0.156	0.164	0.176
ACSCC-20-0.5	0.091	0.131	0.140	0.151	0.161	0.169	0.181
ACSCC-20-0.6	0.108	0.148	0.157	0.168	0.178	0.186	0.198
ACSCC-20-0.8	0.124	0.164	0.173	0.184	0.194	0.202	0.214
ACSCC-20-1.0	0.155	0.195	0.204	0.215	0.225	0.233	0.245

**Table 3:** Water sorptivity of GP-ACSCC mixtures.

Mixture Designation	Sorptivity INDEX (mm/min^1/2^)	
	28 Days	56 Days
ACSCC-00-00	0.24	0.23
ACSCC-05-0.1	0.16	0.12
ACSCC-05-0.2	0.17	0.14
ACSCC-05-0.3	0.18	0.16
ACSCC-05-0.4	0.19	0.17
ACSCC-05-0.5	0.20	0.19
ACSCC-05-0.6	0.21	0.2
ACSCC-05-0.8	0.22	0.21
ACSCC-05-1.0	0.23	0.22
ACSCC-10-0.1	0.17	0.13
ACSCC-10-0.2	0.18	0.15
ACSCC-10-0.3	0.19	0.17
ACSCC-10-0.4	0.20	0.18
ACSCC-10-0.5	0.21	0.20
ACSCC-10-0.6	0.22	0.21
ACSCC-10-0.8	0.23	0.22
ACSCC-10-1.0	0.24	0.23
ACSCC-15-0.1	0.18	0.14
ACSCC-15-0.2	0.19	0.16
ACSCC-15-0.3	0.20	0.18
ACSCC-15-0.4	0.21	0.19
ACSCC-15-0.5	0.22	0.21
ACSCC-15-0.6	0.23	0.22
ACSCC-15-0.8	0.24	0.23
ACSCC-15-1.0	0.25	0.24
ACSCC-20-0.1	0.19	0.15
ACSCC-20-0.2	0.20	0.17
ACSCC-20-0.3	0.21	0.19
ACSCC-20-0.4	0.22	0.20
ACSCC-20-0.5	0.23	0.22
ACSCC-20-0.6	0.24	0.23
ACSCC-20-0.8	0.25	0.24
ACSCC-20-1.0	0.26	0.25

## Data Availability

Request directly to the authors.
